# The effect of varicella-zoster virus reactivation on the long-term outcomes of patients undergoing allogeneic hematopoietic stem cell transplantation

**DOI:** 10.1186/s41043-023-00429-8

**Published:** 2023-10-02

**Authors:** Ping Li, Jingxia Li, Haoyuan Huang, Xiongnong Chen, Yue Lin, Ganlin He, Duorong Xu

**Affiliations:** Department of Haematology, The First Affiliated Hospital of Sun Yat-sen University, No.58 Zhongshan 2nd Road, Guangzhou, 510080 China

**Keywords:** Allogeneic hematopoietic stem cell transplantation, Varicella-zoster virus reactivation, Relapse, Overall survival

## Abstract

**Background:**

A virus infection may lead the body to produce more immune cells of particular types or stimulate the production of new ones, both of which may have anti-leukemic effects. There has been no research on whether immune cells stimulated by varicella-zoster virus (VZV) infection have anti-leukemic effects. The objective of this investigation is to assess the impact of VZV infection on patients' long-term survival following allogeneic hematopoietic stem cell transplantation (allo-HSCT).

**Methods:**

This retrospective study investigated the association between varicella-zoster virus (VZV) reactivation and outcomes in 219 individuals who received allogeneic hematopoietic stem cell transplantation (allo-HSCT) at the Sun Yat-sen University’s First Affiliated Hospital. According to being diagnosed with VZV infection or not, these patients were grouped into two groups. The comparison of cumulative incidence of relapse, non-recurrent mortality, and overall survival (OS) was conducted between the two groups.

**Results:**

Analyzing multivariate data, VZV reactivation was linked to lower relapse incidence in the group containing all individuals (hazard ratio [HR] = 0.27; 95% confidence interval [CI], 0.12–0.64), patients suffering from acute myeloid leukaemia (HR = 0.10; 95% CI, 0.01–0.83), and patients suffering from acute lymphoblastic leukaemia (HR = 0.25; 95% CI, 0.08–0.77). Moreover, VZV reactivation was linked with decreased non-relapse mortality in all individuals (HR = 0.20; 95% CI, 0.05–0.79), but no statistical significance was found for any disease subgroup. Further, VZV reactivation was an independent predictor for improved OS in the group containing all individuals (HR = 0.10; 95% CI, 0.03–0.29), patients suffering from acute myeloid leukaemia (HR = 0.09; 95% CI, 0.01–0.66), and patients suffering from acute lymphoblastic leukaemia (HR = 0.16; 95% CI, 0.04–0.68).

**Conclusion:**

This is the first study to show that VZV reactivation following allo-HSCT is an independent predictor for lower relapse rates and improved OS, providing novel therapeutic approaches to improve patients’ long-term survival following allo-HSCT.

## Background

Allogeneic hematopoietic stem cell transplantation (allo-HSCT) is an efficient therapy that contains the potential to cure hematologic malignancies. Although the incidence of post-transplant relapse is now being lowered by monitoring minimal residual disease [1], creating risk-based preventative strategies [2], and optimizing donor lymphocyte infusions (DLI) [3], relapse continues to be the leading contributory factor to death in individuals following transplantation. Furthermore, because the preconditioning of allo-HSCT leaves the body in a condition of extreme immunodeficiency and years of B- and T-lymphocytopenia after transplantation [4–6], infectious complications remain a difficult challenge to deal with following allo-HSCT.

Recent advances in tumour immunology and evasion mechanisms have rekindled interest in immunotherapies for cancer. Immune cells triggered by an infection may have antitumor effects, particularly in patients with viral infections. This is because natural killer cells and CD8+ T cells, specifically, cytotoxic T cell lymphocytes, are essential components of the immune response to viruses. These cells are also crucial effector cells in immunological reactions against cancer [7–9]. In 2011, a study with 266 patients with acute myeloid leukaemia (AML) published in BLOOD first reported that early replicative cytomegalovirus (CMV) infection was related to a significant reduction in the probability of leukemic relapse after allo-HSCT [10]. In 2013, a study with more than 2000 patients also reported that in AML patients, CMV reactivation was an important protective factor against early relapse following allo-HSCT [11]. Moreover, related articles reported the impact of CMV reactivation on recurrence after allo-HSCT [12–15]. This phenomenon has not only been observed in CMV infection but also the presence of other viruses. Challenor et al. reported a case of stage III Hodgkin lymphoma that reached remission following SARS-CoV-2 infection without corticosteroid or immunochemotherapy [16]. Kamber et al. discovered that varicella-zoster virus (VZV) reactivation was related to a better overall survival rate in patients undergoing autologous stem cell transplant for myeloma [17].

Despite the fact that VZV is a different type of herpes virus, unlike CMV reactivation, no research has been conducted on the association between VZV reactivation and the long-term prognosis of individuals following allo-HSCT. Therefore, the primary intent of this investigation is to assess the connection between VZV reactivation and the cumulative incidence of relapse (CIR), non-recurrent mortality (NRM), and overall survival (OS) in patients after allo-HSCT.

## Methods

### Patients

This retrospective study included 222 patients with AML, myelodysplastic syndrome (MDS), acute lymphoblastic leukaemia (ALL), and chronic myelogenous leukaemia (CML) who underwent their first allo-HSCT at the Sun Yat-sen University’s First Affiliated Hospital between January 1, 2014, and March 31, 2021, with a follow-up cut-off date on June 30, 2021. Patients who had a history of autologous HSCT and those who received their stem cells from both cord blood and peripheral blood were not included in this study. Clinical and demographic information was gathered by reviewing medical records. Clinic visits, telephone conversations with families, and/or reviews of inpatient and outpatient clinical care records were used to gather overall survival data and follow-up information about complications. The First Affiliated Hospital of Sun Yat-sen University’s Ethics Committee gave the study approval, and it was carried out in compliance with the Helsinki Declaration. Two patients received their stem cells from both cord blood and peripheral blood, and one with a history of autologous HSCT were excluded. In total, 219 qualifying patients’ data were incorporated into the final analysis.

### Definition and endpoints

The diagnosis of VZV infection was mainly based on specific clinical manifestations, which were defined by the detection of the VZV antigen or the emergence of typical cutaneous vesicular lesions [18]. VZV reactivation manifested as localized or widespread zoster [18]. The diagnosis of VZV reactivation with unusual symptoms and a suspicious central nervous system infection should be validated by laboratory testing employing VZV-DNA detection or metagenomic sequencing [18]. Acute graft-versus-host disease (aGVHD) and chronic GVHD (cGVHD) were defined using previously reported criteria [19, 20]. Based on the disease risk index (DRI) for allo-HSCT, subjects were categorized into three OS risk cohorts, i.e., favourable, intermediate, and adverse [21]. Conditioning regimens were categorized into two types, based on previously published consensus criteria, i.e., myeloablative conditioning (MAC) and non-myeloablative conditioning (NMAC) [22]; in this study, the latter included non-myeloablative and reduced intensity conditioning. CMV reactivation was referred to any two consecutive CMV-DNA ≥ 500 copies/mL in the blood by using quantitative real-time polymerase chain reaction detection [23].

The primary endpoint of this study was CIR stratified by VZV reactivation. The secondary endpoints were the NRM and OS stratified by VZV reactivation. In this study, relapse after allo-HSCT referred to morphological relapse at any site as determined by the standard criteria. NRM was defined as mortality resulting from any reason other than relapse and was regarded as a competing risk for recurrence. OS was characterized as an interval from transplantation to the last follow-up date or death from any cause. Long-term survival was defined as OS ≥ 3 years. The starting point of follow-up was the date of allo-HSCT.

### Antiviral prophylaxis and treatment of VZV reactivation

Antiviral drugs should be administered prophylactically or preventively after allo-HSCT. To prevent infection of herpes simplex virus types 1 and 2, as well as varicella-zoster virus, valacyclovir was administered to these patients at least 6- 12 months after transplantation, and prolonged treatment course depended on the circumstances. If CMV DNAemia arises, replace valaciclovir with ganciclovir or foscarnet until DNA PCR for CMV is negative. For patients with VZV reactivation, valacyclovir (1000 mg 3 times daily) was given to these patients for 7 days [24], and the course of treatment could be extended if required.

### Statistical analysis

Normally distributed measurement data were expressed as mean ± standard deviation, and the relevant data was analyzed using the independent samples t-test. The chi-square test was conducted to analyze categorical data that met the normal distribution. Non-normally distributed measurements and categorical data were analyzed using the Mann–Whitney U non-parametric test. The CIR and NRM stratified by VZV reactivation were calculated using Gray’s test, with NRM and relapse acting as competing risks. Fine and Gray’s proportional hazard model was employed to examine competing risks in a regression setting. The Kaplan–Meier technique was used to estimate OS probability, and the log-rank test was used to compare estimates. Hazard ratios (HRs) were estimated from the Cox regression models. Covariates included age of patients and donors, donor/recipient sex (mismatch vs. match), DRI (high risk or intermediate risk vs. low risk), HLA matching and donor relation, conditioning regimen (NMAC vs. MAC), the use of anti-thymocyte globulin (ATG) in the conditioning regimen (yes vs. no), DLI after transplantation (yes vs. no), VZV reactivation after transplant (yes vs. no), degrees of aGVHD (grade 2–4 vs grade 0–1), the occurrence of cGVHD (yes vs. no), and CMV reactivation after transplant (yes vs. no). All of the relevant risk factors were found using a forward-stepwise model selection method. Risk factors with *P* ≤ 0.05 in univariate models were retained in the final model, and VZV reactivation was forced into the final model. The significance threshold for all bilateral tests was established at *P* < 0.05. The SPSS Statistics (v.25.0, SPSS Inc., Chicago, IL, USA) and R 4.1.2 package cmprsk (v.4.1.2, https://www.r-project.org/) software were used.

## Results

### Patient characteristics

The demographic and clinical data of all patients included in this study are summarized in Table 1. Among the 219 qualifying patients, 106 were diagnosed with AML, 94 with ALL, 8 with CML, and 11 with MDS. Due to the limited number of cases, data from CML and MDS patients were not evaluated individually. In the entire cohort, almost two-thirds of the patients (62.6%) were male, having a median age of 32 years (range, 14–58 years). Similarly, most donors were male, with a median age of 31 years (range, 11–62 years). Nearly all patients used MAC as a transplantation conditioning regimen, and 77.2% used ATG during the pretreatment phase; this was because 56.6% of patients received transplants from HLA-mismatched related donors and 7.8% got transplants from unrelated donors. The median follow-up duration for all patients was 642 days, with a 5-year OS of 60.2% (95% confidence interval [CI], 52.6–68.8%).

**Table 1:** Characteristics of patients classified by VZV reactivation

	All patients	Patients without VZV reactivation	Patients with VZ reactivation	P Value
No. of patients	219	169	50	
Patient age (y)				0.188
Median (range)	32 (14- 58)	31 (14- 58)	35.5 (16- 57)	
Patient sex				0.554
Female	82 (37.4%)	61 (36.1%)	21 (42.0%)	
Male	137 (62.6%)	108 (63.9%)	29 (58.0%)	
Disease type				0.914
AML	106 (48.4%)	82 (48.5%)	24 (48.0%)	
ALL	94 (42.9%)	73 (43.2%)	21 (42.0%)	
Others*	19 (8.68%)	14 (8.28%)	5 (10.0%)	
Disease risk index				0.954
Low	2 (0.91%)	2 (1.18%)	0 (0.00%)	
Intermediate	188 (85.8%)	142 (84.0%)	44 (88.0%)	
High	29 (13.2%)	23 (13.6%)	6 (12.0%)	
Donor type				0.389
Matched related	78 (35.6%)	61 (36.1%)	17 (34.0%)	
Mismatched related	124 (56.6%)	94 (55.6%)	30 (60.0%)	
Matched unrelated	9 (4.11%)	6 (3.55%)	3 (6.00%)	
Mismatched unrelated	8 (3.65%)	8 (4.73%)	0 (0.00%)	
Sex matched				0.276
Matched	109 (49.8%)	88 (52.1%)	21 (42.0%)	
Mismatched	110 (50.2%)	81 (47.9%)	29 (58.0%)	
Donor sex				0.341
Female	69 (31.5%)	50 (29.6%)	19 (38.0%)	
Male	150 (68.5%)	119 (70.4%)	31 (62.0%)	
Donor age (y)				0.516
Median (range)	31 (11- 62)	30 (11- 62)	33 (14- 60)	
Conditioning regimen				1.000
NMAC	1 (0.46%)	1 (0.59%)	0 (0.00%)	
MAC	214 (99.5%)	168 (99.4%)	50 (100%)	
ATG				0.463
No	50 (22.8%)	41 (24.3%)	9 (18.0%)	
Yes	169 (77.2%)	128 (75.7%)	41 (82.0%)	
DLI				0.870
No	189 (86.3%)	145 (85.8%)	44 (88.0%)	
Yes	30 (13.7%)	22 (14.2%)	6 (12.0%)	
aGvHD				0.850
Grade: 0–1	123 (56.2%)	96 (56.8%)	27 (54.0%)	
Grade: 2–4	96 (43.8%)	73 (43.2%)	23 (46.0%)	
cGvHD				0.113
No	120 (54.8%)	98 (58.0%)	22 (44.0%)	
Yes	99 (45.2%)	71 (42.0%)	28 (56.0%)	
CMV reactivation				0.285
No	91 (41.6%)	74 (43.8%)	17 (34.0%)	
Yes	128 (58.4%)	95 (56.2%)	33 (66.0%)	

MA myeloablative conditioning; NMAC, non-myeloablative conditioning; ATG, antithymocyte globulin; DLI, donor lymphocyte infusion; GvHD, graft versus host disease; CMV reactivation, cytomegalovirus reactivation

*Others indicate 8 CML patients and 11 MDS patients

### VZV reactivation

Of all the included patients, 50 (22.8%) experienced VZV reactivation after allo-HSCT. As shown in Table 1, 96 patients experienced grade 2–4 aGVHD, with 23 instances (46.0%) occurring in patients with VZV reactivation and 73 instances (43.2%) occurring in patients without VZV reactivation (*P* = 0.850). Additionally, 169 patients used ATG, with 41 (82.0%) patients with VZV reactivation and 128 (75.7%) patients without VZV reactivation (*P* = 0.463). Furthermore, 99 patients had cGVHD, with 28 instances (56.0%) occurring in patients with VZV reactivation and 71 instances (42.0%) occurring in patients without VZV reactivation (*P* = 0.113). No significant variations in the distribution of age, gender, disease type, DRI, pretreatment regimen, DLI usage, or incidence of CMV reactivation were found between the two groups. Between the two groups, the initial features were comparable (Table 1).

### VZV reactivation and relapse

**Fig. 1:**
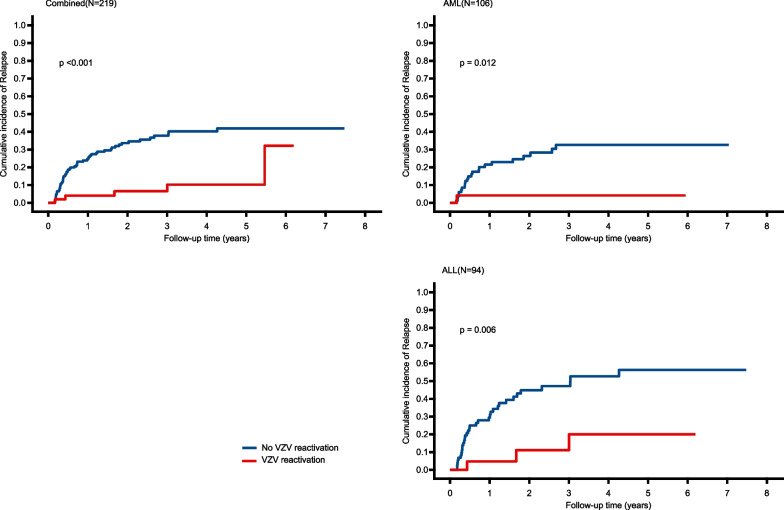
Cumulative incidence of relapse stratified by VZV reactivation after allo-HSCT

**Fig. 2:**
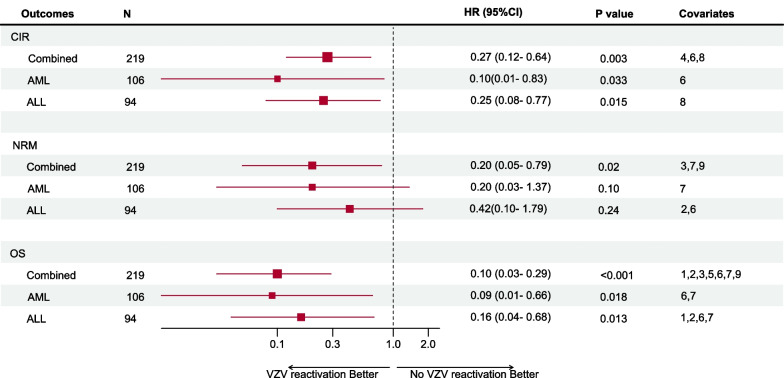
HR and 95% CI from multivariable models evaluating VZV reactivation as a risk factor for relapse, non-relapse mortality and overall survival (OS) after allo-HSCT. Covariates: 1, donor age; 2, donor sex; 3, donor type (Mismatched related vs. Matched related, Matched unrelated vs. Matched related, or Mismatched unrelated vs. Matched related); 4, disease type (ALL vs. AML, others (CML/MDS) vs. AML); 5, High vs. low, intermediate; 6, donor lymphocyte infusion (yes vs. no); 7, acute GVHD (grade 3–4 vs. 0–2); 8, chronic GVHD (yes vs. no); 9, CMV reactivation (yes vs. no)

Upon the end of the follow-up period (June 2021), 64 of the 219 patients (29.2%) relapsed following transplantation. As shown in Fig. 1, the CIR was 4.0% and 6.6% in cases with VZV reactivation, compared with 26.7% and 42.6% for those without VZV reactivation at 1 year and 3 years after transplantation, respectively (*P* < 0.001) (Fig. 1). In the univariate competing-risks regression analysis, significantly fewer relapses occurred in patients who had VZV reactivation compared with those who did not. After adjusting for other covariates in multivariate competing-risks regression analyses in the combined cohort (Fig. 2), VZV reactivation was still recognized as an independent factor linked to a lower incidence of relapse (hazard ratio [HR], 0.27; 95% CI, 0.12–0.64).

Subgroup analyses were conducted according to disease types. Owing to the low number of MDS and CML patients, they were not evaluated separately but were included in the analysis as part of the entire cohort. As shown in Fig. 1, the CIR was 4.2% and 4.2% when VZV reactivation was present, and 23.6% and 36.7% when VZV reactivation was not present at 1 year and 3 years after the transplantation in AML patients (*P* = 0.012). The CIR was 4.8% and 11.1% when VZV reactivation was present, and 31.3% and 51.8% when VZV reactivation was not present at 1 year and 3 years after the transplantation in ALL patients (*P* = 0.006). After adjusting for other covariates in multivariate competing-risks regression analyses by disease type (Fig. 2), the position of VZV reactivation occurring as a beneficial independent variable associated with relapse incidence remained unchanged in AML patients (HR, 0.10; 95% CI, 0.01 to 0.83) or ALL patients (HR, 0.25; 95% CI, 0.08 to 0.77).

### VZV reactivation and NRM and OS

**Fig. 3:**
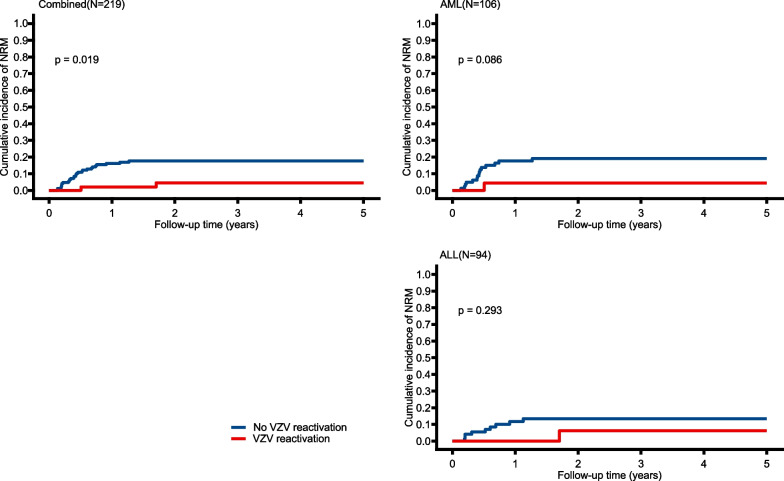
Cumulative incidence of non-relapse mortality stratified by VZV reactivation after allo-HSCT

At the time of data cut-off, 148 patients were still alive and 71 had died, with 41 deaths occurring from disease relapse and 30 from NRM. The 1- and 3-year NRM were 17.1% and 19.0% in patients without VZV, compared with 2.0% and 4.6% for patients with VZV reactivation (*P* = 0.019) (Fig. 3). Confirmed by multivariable competing-risks regression analyses, the occurrence of VZV reactivation was linked to an 80% decreased NRM in the group containing all patients (HR, 0.20; 95% CI, 0.05 to 0.79). However, for subgroup analysis, based on disease type, the statistical significance of the effect of VZV reactivation on NRM was not observed in both AML patients and ALL patients (Fig. 2).

**Fig. 4:**
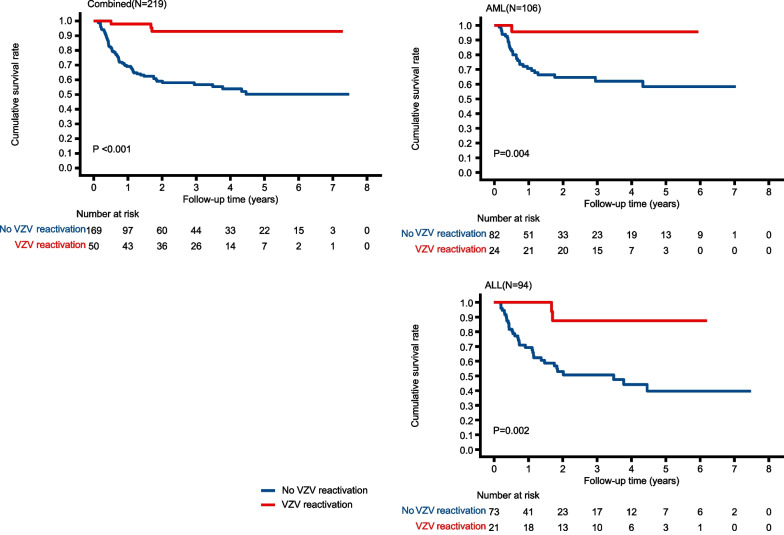
Cumulative rate of OS stratified by VZV reactivation after allo-HSCT

In the entire cohort, the estimated 1- and 3-year estimated rates of OS were 75.9% and 65.4%, respectively. When stratified by VZV reactivation, the estimated rate of OS was 69.1% and 56.7% in patients without VZV reactivation, compared with 98.0% and 92.9% for those with VZV reactivation at 1 year and 3 years after the transplantation in the combined group (*P* < 0.001) (Fig. 4). For disease-type-based subgroup analysis, the estimated rates of OS were 70.7% and 62.0% in patients without VZV reactivation, compared with 95.7% and 95.7% for those with VZV reactivation at 1 year and 3 years after transplantation in AML patients (*P* = 0.004) (Fig. 4). The estimated rates of OS were 69.3% and 50.7% in patients without VZV reactivation, compared with 100% and 87.5% for those with VZV reactivation at 1 year and 3 years after transplantation in ALL patients (*P* = 0.002) (Fig. 3). Confirmed by the multivariate Cox regression analysis, VZV reactivation was an independent predictor for improved OS in the group containing all individuals (HR, 0.10; 95% CI, 0.03 to 0.29), AML patients (HR, 0.09; 95% CI, 0.01 to 0.66), or ALL patients (HR, 0.16; 95% CI, 0.04 to 0.68) (Fig. 2).

## Discussion

This investigation intended to explore the relationship between VZV reactivation and patients’ long-term prognoses following allo-HSCT, including CIR, NRM, and OS. In the combined cohort, VZV reactivation was significantly linked with decreased relapse, decreased NRM, and increased OS. The multivariate regression analysis confirmed that VZV reactivation was an independent protective predictor of CIR, NRM, and OS. For disease-type-based subgroup analysis, whether in AML or ALL patients, the multivariate regression analysis confirmed that VZV reactivation was still an independent protective predictor of CIR and OS. Our findings provide a way to lessen recurrence following allo-HSCT.

The prognosis could be influenced by the patient's pre-transplant status, the transplant procedure, post-transplant complications, and donor status. In these two groups, the variations in patient demographic features, disease type, DRI, conditioning regimen, the incidence of aGVHD, the incidence of cGVHD, the incidence of CMV DNAemia and donor demographic characteristics were not statistically significant. Also, after multivariate analysis, VZV reactivation proved to remain significant and was the strongest risk factor. This boosted the reliability of the findings of this study.

Whether VZV reactivation substantially contributes to the graft-versus-leukemic effect is unknown. However, it is worth investigating whether cellular immunity induced by VZV reactivation has an anti-leukemic effect.

Firstly, the reactivation of CMV, another herpes virus, may induce cellular anti-leukemic immunity [25]. Much has been learned about the mechanisms of anti-leukemic effects induced by CMV reactivation after allo-HSCT, which propose that circulating Vδ2^neg^γδ T-cells expand strongly and permanently, and these cells recognize leukemic blasts via their TCR, and CD8αα probably serves as a co-receptor in antigen recognition [10, 15, 26]. It has been well documented that post-transplant or immunocompromised patients with VZV reactivation had markedly decreased CD4+ T cells and surface CD28 expression but increased CD8 + T cells [27, 28]. This immunological response to VZV infection was not weakened by acyclovir treatment [29]. Moreover, CD8+ T cells, specifically, cytotoxic T cell lymphocytes, are essential components in the immune response to viruses, and these cells are also crucial effector cells in the immunological reactions against cancer [7–9].

Secondly, CMV infection may increase NRM which counteracts the benefit of the graft-versus-leukemic effect, and the graft-versus-leukemic effect may be influenced by several factors. A Japanese study involving 3,539 patients who underwent allo-HSCT demonstrated that CMV reactivation was significantly linked with reduced disease recurrence only in AML patients, but not in ALL, CML, or MDS patients. Additionally, CMV reactivation was strongly associated with increased NRM and overall mortality [15, 30]. But in the combined cohort in our study, VZV reactivation was not only significantly related to decreased CIR, but also decrease NRM. Due to the limited number of patients in this study, a disease-based subgroup analysis solely examined the AML patients and ALL patients, which revealed that the beneficial impact of VZV reactivation on disease recurrence and OS was not restricted to AML patients. Meanwhile, the Varicella-zoster virus serves as the only human herpes virus with a highly effective vaccine [18]. Unlike most vaccinations, which predominantly produce antibodies for protection, the varicella vaccine primarily produces cellular immunity [31]. Additionally, the Oka VZV vaccine strain could potentially elicit decades-long antiviral antibodies, lymphoproliferative responses, and cytotoxic T-cell responses. Furthermore, existing studies indicated that the inactivated VZV vaccine was safe for patients with haematological malignancies who had undergone chemotherapy or hematopoietic stem cell transplantation [32, 33]. Moreover, a credible assessment approach of VZV-specific cell-mediated immunity has been proposed to quantify the expression of the ifn-γ gene in 24-h-stimulated whole blood [34], which could aid in understanding the cellular immunity of VZV reactivation. However, VZV reactivation will damage the quality of life for the patients. Tatebe et al. pointed out that VZV reactivation following HSCT in children could be prevented by using low-dose acyclovir [35]. Therefore, as advised in the guidelines, routine prophylactic antiviral therapy should be provided to all patients following allo-HSCT. Our ultimate objective is to identify the varicella-zoster virus antigen, which could activate particular immune cells with antileukemic properties. These provide opportunities for the development of VZV-related oncology vaccines.

Additionally, even in AML patients, Bao et al. discovered that in the ATG-using cohort, T-cell depletion diminished the anti-leukaemia effects induced by CMV reactivation [36]. But in patients with VZV reactivation in this study, more patients used ATG than patients without, although the lack of statistical significance, which implied that VZV reactivation may result in robust anti-leukaemia effects, and the anti-leukaemia effects may be less affected by other factors.

Therefore, it is plausible and worthy to hypothesize that VZV reactivation may directly contribute to anti-leukemic effects. To confirm this, prospective studies are urgently needed to assess the enduring immunological response of patients with VZV reactivation following allo-HSCT. It is reasonable to postulate that VZV reactivation directly contributes to the substantial long-term antileukemia effect reported in this study.

## Conclusions

In conclusion, after adjusting for confounding variables, this study revealed that VZV reactivation was linked to a significant decrease in CIR and NRM in patients following allo-HSCT and was an independent predictor of an excellent OS rate. This phenomenon of reduced leukaemia recurrence should be validated by several prospective studies, and the mechanisms underlying this anti-leukemic impact must also be confirmed by multiple prospective and experimental research.

## Data Availability

Sending an email to the corresponding author will give you access to the raw data.

## Abbreviations

VZV: Varicella-zoster virus
allo-HSCT: Allogeneic hematopoietic stem cell transplantation
HR: Hazard ratio
CI: Confidence interval
ALL: Acute lymphoblastic leukaemia
AML: Acute myeloid leukaemia patients
DLI: Donor lymphocyte infusions
CMV: Cytomegalovirus
CIR: Cumulative incidence of relapse
NRM: Non-recurrent mortality
OS: Overall survival
MDS: Myelodysplastic syndrome
CML: Chronic myelogenous leukaemia
aGVHD: Acute graft-versus-host disease
cGVHD: Chronic GVHD
DRI: Disease risk index
MAC: Myeloablative conditioning
NMAC: Non-myeloablative conditioning
ATG: Antithymocyte globulin

## References

[1] Qin YZ, Chen Y, Xu LP (2018). Outcome and minimal residual disease monitoring in patients with t(16;21) acute myelogenous leukemia undergoing allogeneic hematopoietic stem cell transplantation. Biol Blood Marrow Transplant J Am Soc Blood Marrow Transplant.

[2] Burchert A, Bug G, Fritz LV (2020). Sorafenib maintenance after allogeneic hematopoietic stem cell transplantation for acute myeloid leukemia with FLT3-internal tandem duplication mutation (SORMAIN). J Clin Oncol Off J Am Soc Clin Oncol.

[3] Yan CH, Liu DH, Liu KY (2012). Risk stratification-directed donor lymphocyte infusion could reduce relapse of standard-risk acute leukemia patients after allogeneic hematopoietic stem cell transplantation. Blood.

[4] Sutrave G, Blyth E, Gottlieb DJ (2017). Cellular therapy for multiple pathogen infections after hematopoietic stem cell transplant. Cytotherapy.

[5] Onozawa M, Hashino S, Takahata M (2006). Relationship between preexisting anti-varicella-zoster virus (VZV) antibody and clinical VZV reactivation in hematopoietic stem cell transplantation recipients. J Clin Microbiol.

[6] Stadtmauer EA, Sullivan KM, Marty FM (2014). A phase 1/2 study of an adjuvanted varicella-zoster virus subunit vaccine in autologous hematopoietic cell transplant recipients. Blood.

[7] Deets KA, Vance RE (2021). Inflammasomes and adaptive immune responses. Nat Immunol.

[8] Kahan SM, Bakshi RK, Ingram JT (2022). Intrinsic IL-2 production by effector CD8 T cells affects IL-2 signaling and promotes fate decisions, stemness, and protection. Sci Immunol..

[9] Kim TS, Shin EC (2019). The activation of bystander CD8(+) T cells and their roles in viral infection. Exp Mol Med.

[10] Elmaagacli AH, Steckel NK, Koldehoff M (2011). Early human cytomegalovirus replication after transplantation is associated with a decreased relapse risk: evidence for a putative virus-versus-leukemia effect in acute myeloid leukemia patients. Blood.

[11] Green ML, Leisenring WM, Xie H (2013). CMV reactivation after allogeneic HCT and relapse risk: evidence for early protection in acute myeloid leukemia. Blood.

[12] Cichocki F, Cooley S, Davis Z (2016). CD56dimCD57+NKG2C+ NK cell expansion is associated with reduced leukemia relapse after reduced intensity HCT. Leukemia.

[13] Koldehoff M, Ross SR, Dührsen U, Beelen DW, Elmaagacli AH (2017). Early CMV-replication after allogeneic stem cell transplantation is associated with a reduced relapse risk in lymphoma. Leuk Lymphoma.

[14] Manjappa S, Bhamidipati PK, Stokerl-Goldstein KE (2014). Protective effect of cytomegalovirus reactivation on relapse after allogeneic hematopoietic cell transplantation in acute myeloid leukemia patients is influenced by conditioning regimen. Biol Blood Marrow Transplant.

[15] Takenaka K, Nishida T, Asano-Mori Y (2015). Cytomegalovirus reactivation after allogeneic hematopoietic stem cell transplantation is associated with a reduced risk of relapse in patients with acute myeloid leukemia who survived to day 100 after transplantation: the Japan society for hematopoietic cell transplantation transplantation-related complication working group. Biol Blood Marrow Transplant.

[16] Challenor S, Tucker D (2021). SARS-CoV-2-induced remission of Hodgkin lymphoma. Br J Haematol.

[17] Kamber C, Zimmerli S, Suter-Riniker F (2015). Varicella zoster virus reactivation after autologous SCT is a frequent event and associated with favorable outcome in myeloma patients. Bone Marrow Transplant.

[18] Gershon AA, Breuer J, Cohen JI (2015). Varicella zoster virus infection. Nat Rev Dis Primers.

[19] Sung AD, Chao NJ (2013). Concise review: acute graft-versus-host disease: immunobiology, prevention, and treatment. Stem Cells Transl Med.

[20] Lee SJ (2017). Classification systems for chronic graft-versus-host disease. Blood.

[21] Armand P, Kim HT, Logan BR (2014). Validation and refinement of the disease risk index for allogeneic stem cell transplantation. Blood.

[22] Bacigalupo A, Ballen K, Rizzo D (2009). Defining the intensity of conditioning regimens: working definitions. Biol Blood Marrow Transplant.

[23] Ljungman P, Boeckh M, Hirsch HH (2017). Definitions of cytomegalovirus infection and disease in transplant patients for use in clinical trials. Clin Infect Dis.

[24] Werner RN, Nikkels AF, Marinović B (2017). European consensus-based (S2k) Guideline on the Management of Herpes Zoster - guided by the European Dermatology Forum (EDF) in cooperation with the European Academy of Dermatology and VENEREOLOGY (EADV), part 2: treatment. J Eur Acad Dermatol Venereol.

[25] Foley B, Cooley S, Verneris MR (2012). Cytomegalovirus reactivation after allogeneic transplantation promotes a lasting increase in educated NKG2C+ natural killer cells with potent function. Blood.

[26] Yoon JH, Lee S, Kim HJ (2016). Impact of cytomegalovirus reactivation on relapse and survival in patients with acute leukemia who received allogeneic hematopoietic stem cell transplantation in first remission. Oncotarget.

[27] Vermont CL, Jol-van der Zijde EC, Hissink Muller P (2014). Varicella zoster reactivation after hematopoietic stem cell transplant in children is strongly correlated with leukemia treatment and suppression of host T-lymphocyte immunity. Transpl Infect Dis Off J Transplant Soc..

[28] Murata K, Hoshina T, Onoyama S (2020). Reduction in the number of Varicella-Zoster virus-specific T-cells in immunocompromised children with varicella. Tohoku J Exp Med.

[29] Kumagai T, Kamada M, Igarashi C (1999). Varicella-zoster virus-specific cellular immunity in subjects given acyclovir after household chickenpox exposure. J Infect Dis.

[30] Teira P, Battiwalla M, Ramanathan M (2016). Early cytomegalovirus reactivation remains associated with increased transplant-related mortality in the current era: a CIBMTR analysis. Blood.

[31] Beals CR, Railkar RA, Schaeffer AK (2016). Immune response and reactogenicity of intradermal administration versus subcutaneous administration of varicella-zoster virus vaccine: an exploratory, randomised, partly blinded trial. Lancet Infect Dis.

[32] Mullane KM, Morrison VA, Camacho LH (2019). Safety and efficacy of inactivated varicella zoster virus vaccine in immunocompromised patients with malignancies: a two-arm, randomised, double-blind, phase 3 trial. Lancet Infect Dis.

[33] Winston DJ, Mullane KM, Cornely OA (2018). Inactivated varicella zoster vaccine in autologous haemopoietic stem-cell transplant recipients: an international, multicentre, randomised, double-blind, placebo-controlled trial. Lancet.

[34] Boccard M, Conrad A, Mouton W (2022). A simple-to-perform ifn-γ mRNA gene expression assay on whole blood accurately appraises varicella zoster virus-specific cell-mediated immunity after allogeneic hematopoietic stem cell transplantation. Front Immunol.

[35] Tatebe Y, Ushio S, Esumi S (2022). Low-dose acyclovir for prophylaxis of varicella-zoster virus reactivation after hematopoietic stem cell transplantation in children. Pediatr Blood Cancer.

[36] Bao X, Zhu Q, Xue S (2016). Cytomegalovirus induces strong antileukemic effect in acute myeloid leukemia patients following sibling HSCT without ATG-containing regimen. Am J Transl Res.

